# Single-Shot Waterless Low-Profile Photoacoustic System: Near-Field Volumetric Imaging In Vivo for Blood Vessels Based on Capacitive Micromachined Ultrasonic Transducer (CMUT)

**DOI:** 10.3390/s19050995

**Published:** 2019-02-26

**Authors:** Won Young Choi, Young Hun Kim, Hyeong Geun Jo, Joo Young Pyun, Soo Won Kwon, Kwan Kyu Park

**Affiliations:** Department of Mechanical Convergence Engineering, Hanyang University, Seoul 04763, Korea; cwy1533@hanyang.ac.kr (W.Y.C.); jason401@hanyang.ac.kr (Y.H.K.); sjdf5702@hanyang.ac.kr (H.G.J.); juyoung@hanyang.ac.kr (J.Y.P.); rnjstndnjs@hanyang.ac.kr (S.W.K.)

**Keywords:** photoacoustic, near field imaging, CMUT, single shot imaging

## Abstract

Intensive research on photoacoustics (PA) for imaging of the living human body, including the skin, vessels, and tumors, has recently been conducted. We propose a PA measurement system based on a capacitive micromachined ultrasonic transducer (CMUT) with waterless coupling, short measurement time (<1 s), backward light irradiation, and a low-profile ultrasonic receiver unit (<1 cm). We fabricate a 64-element CMUT ring array with 6.2 mm diameter and 10.4 MHz center frequency in air, and 100% yield and uniform element response. To validate the PA tissue characterization, we employ pencil lead and red ink as solid and liquid models, respectively, and a living body to target moles and vessels. The system implements a near-field imaging system consisting of a 6 mm polydimethylsiloxane (PDMS) matching layer between the object and CMUT, which has a 3.7 MHz center frequency in PDMS. Experiments were performed in a waterless contact on the PDMS and the laser was irradiated with a 1 cm diameter. The experimental results show the feasibility of this near-field PA imaging system for position and depth detection of skin, mole, vessel cells, etc. Therefore, a system applicable to a low-profile compact biomedical device is presented.

## 1. Introduction

Recently, photoacoustic imaging (PAI) in soft tissue has been studied extensively in the context of biomedical imaging. Photoacoustics (PA) combines the physics of optical and ultrasound imaging and exploits the PA effect, in which a photon incident on tissue generates scattered ultrasonic waves through optical absorption and thermal expansion. Compared with optical imaging techniques, PAI provides deeper penetration and superior spatial resolution and contrast, as determined by the ultrasonic transducer (UT) specifications such as the center frequency, cell size and pitch, element size and pitch, and receiving sensitivity [1,2]. Thus, PAI is useful for cell, tissue, and organ imaging of living biological structures. It is mainly used to detect tumors and vessels, which have higher optical absorption and thermal expansion coefficients than surrounding tissue [3,4,5].

Most previous PAI studies have targeted spatial resolution and contrast improvement. PA microscopy (PAM) is an excellent method for obtaining high resolution and high contrast. Optical resolution PA microscopy (ORPAM) uses the optical focus to emit ultrasonic waves at the focal point of light sources. Further, as acoustic signals are generated at a focal spot, high resolution and high contrast can be achieved [6,7]. Thus, acoustic resolution PA microscopy (ARPAM) has been developed, in which the spatial resolution is primarily determined by the focused transducer aperture and wavelength. A transducer with low F-number and high frequency can produce high resolution [8,9]. These PAM techniques can yield high resolution and high contrast, but have long operation times because mechanical scanning is required. PA tomography (PAT) can provide multi-scale and multi-contrast biological structure imaging with high spatial resolution [10,11,12], high contrast, and satisfactory imaging depth. However, PAT is a section imaging method; thus, mechanical scanning and a bulky setup are needed to determine the object volumetric structure. Previously, Lin et al. [13] implemented a single-breath-hold system [13] targeting the breast. However, the designed system is bulky.

In this article, motivated by ultrasound medical imaging, we demonstrate PA volumetric imaging using a capacitive micromachined UT (CMUT), which can be applied in compact devices such as smart watches and bands. A 2D transducer array is highly desirable for realization of 3D volumetric PAI. Previous studies on PA volumetric imaging have been performed, but the system application is limited by far field imaging, the immersion method, and bulkiness [14,15,16]. Here, we fabricate a 64-channel CMUT ring array and create a hole inside the CMUT elements to form an optical path for the light source integrated behind the CMUT. The employed backward-mode light irradiation provides more uniform illumination and ultrasonic wave emission in the near field. Therefore, the acoustic sensing system presented in this work has the following advantages: waterless coupling, backward-mode laser irradiation, a low-profile ultrasonic receiver unit (<1 cm), and short measurement intervals (<1 s). Thus, application of this PA system in various compact devices is beneficial.

## 2. Imaging System and Method

### 2.1. Construction System and CMUT Fabrication

**Figure 1:**
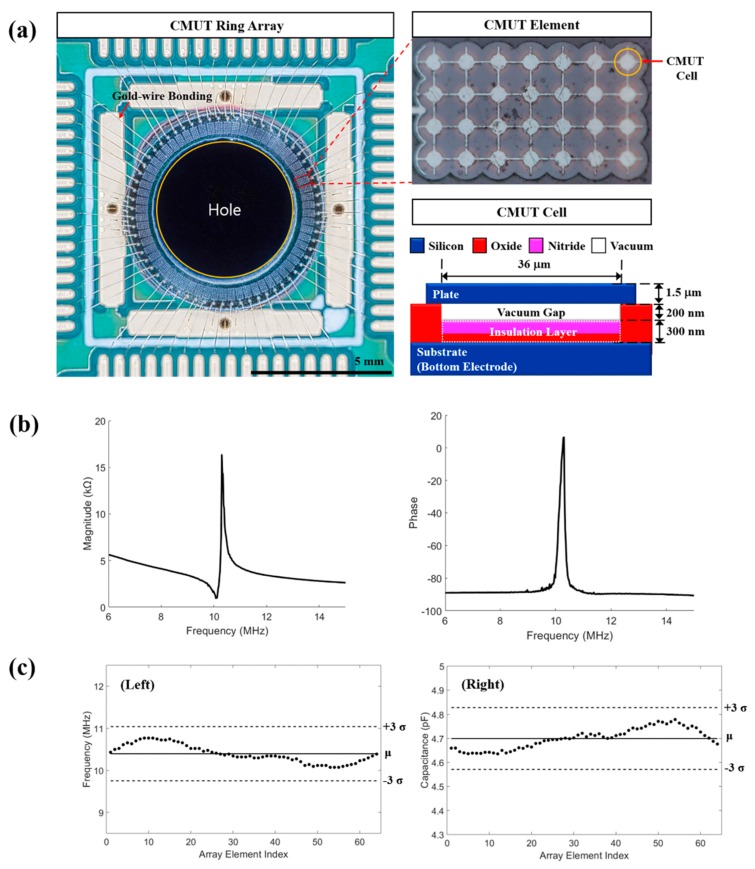
(**a**) Capacitive micromachined ultrasonic transducer (CMUT) ring array bonded with gold wire, closed CMUT element, and cross-sectional schematic of CMUT cell. (**b**) Measured magnitude and phase of electrical impedance (**c**) Measured resonance frequency in air (left) and capacitance (right) of elements.

In the PAI method, lights are used as acoustic signal generators and UTs as receivers. The acoustic signal generation characteristics, such as the radiated light direction, intensity, and uniformity, and the acoustic signal receiving characteristics, such as the receiving sensitivity, spatial resolution, and grating lobe effects, are all important. Calibration of the light path to determine the illumination uniformity and intensity of the generated acoustic signal is the key to constructing an excellent PA system. Fabrication of transparent UTs [17] and the application of optical fibers [18] and optical-acoustic objectives [19] have been implemented to achieve backward-mode light irradiation. To implement the backward-mode receiving system in this study, we fabricated a ring-type CMUT array with a hole inside the elements, as shown in Figure 1. The hole to secure the light path was created by laser cutting the central part of the silicon substrate, which did not affect the electrical and mechanical characterization of the transducer. The laser-cut CMUT ring array had outer and inner diameters of 6.2 and 4.5 mm, respectively.

The UT used as the receiver is as important as the light irradiation. A CMUT has advantages such as wide bandwidth, ease of array fabrication, potential for integration with electronics, and high receiving sensitivity [20]. In addition, a CMUT can be applied in compact devices, as it is fabricated via a micro-electro-mechanical systems process. Use of a 2D dense array is optimal for implementation of a 3D volumetric imaging system; however, high yield in a complicated fabrication process and the control of many channels are challenging tasks. Here, we fabricated a 64-element 2D CMUT ring array with 100% yield and uniform element response. As shown in Figure 1a, the CMUT consisted of a silicon upper plate, a vacuum gap with a silicon nitride plate beneath it, and a silicon oxide plate acting as insulation at the bottom. It was fabricated via direct wafer bonding with local oxidation of silicon [21]. This CMUT was designed for 10.4 MHz center frequency in air and 50 V collapse voltage. Center frequency was determined by plate thickness and radius, as well as, collapse voltage was determined by insulation layer permittivity and thickness, vacuum gap height, and plate radius. The plate was designed of single-crystal silicon with 1.5 μm thickness, 18 μm radius to meet the target center frequency. In addition, parameters such as the 200 nm vacuum gap height and the 300 nm insulation layer thickness considering effective capacitance were applied to each cell for the collapse voltage. To arrange the elements in a circular shape of 6.2 mm diameter, each element was designed as follows: 28 cells arranged in 4 × 7 rectangular distributions with 41 μm cell-to-cell pitch, 258 μm element-to-element pitch (0.95λ), and 159 μm × 282 μm element size. As the electrical impedance indicates the expected mechanical behavior of the CMUT, the impedance of each individual element was measured by an impedance analyzer (HP 4194A, Hewlett Packard Inc., Palo Alto, CA, USA). Figure 1b shows measured electrical impedance results with 40 V bias voltage, which was applied to the experiment. Figure 1c shows the measurement results. The mean value of the device capacitance was 4.70 pF with a standard deviation (SD) of 0.042 pF. The mean value of the resonance frequency in air was 10.4 MHz with a 0.215-MHz SD. Thus, this CMUT device had 100% functional element yield and excellent uniformity.

### 2.2. Construction of Low Profile CMUT Photoacoustic System

**Figure 2:**
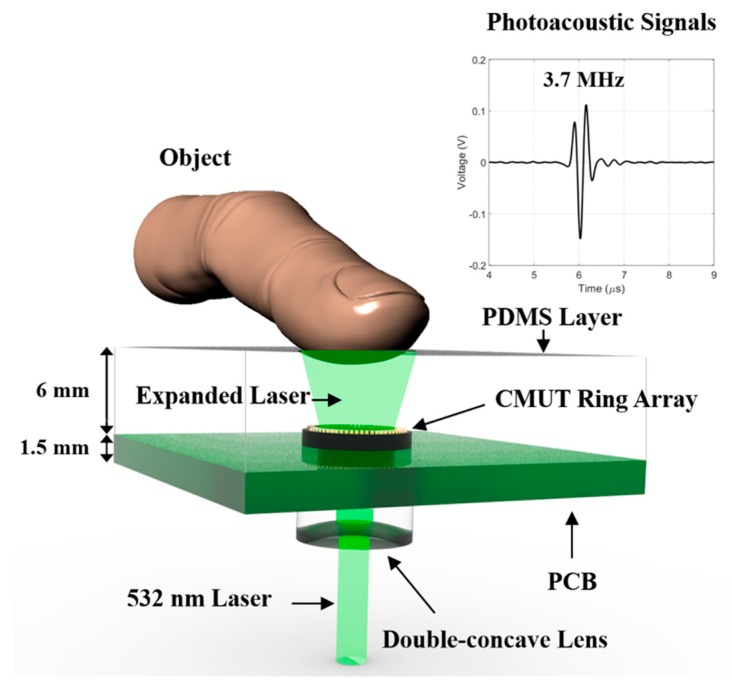
Overall configuration of the photoacoustic (PA) system with a-scan data detected acoustic signals.

In this work, as shown in Figure 2, the overall integrated circuit system was designed focusing on a low-profile dry contact approach. The device had a polydimethylsiloxane (PDMS) layer of 6 mm thickness, with a printed circuit board (PCB) of 1.5 mm thickness, and a lens with 2.29 mm edge thickness. Each CMUT element was bonded to the PCB with gold wire for electrical connection. The custom-designed board consisted of a receiving signal amplifier fabricated using an op-amp (OPA820, Texas Instruments Inc., Dallas, TX, USA), connection lines supplying direct current to the CMUT for the operating receiver mode, and the electrical connections of the individual elements. All elements were activated in the experiment. The circuit could amplify the acoustic signals by 30 dB equally for each element. PDMS (Sylgard 184, Dow Corning Co., Midland, MI, USA), which has 0.982 g/ml density and 1000-m/s speed of sound, was molded on the front side of the CMUT to allow device protection and subject contact. The transparent PDMS used in the device had very low optical absorption; thus, it was an excellent material for use in an optical pathway. To expand the light illumination, we attached a double-concave lens (Edmund Optics Inc., Barrington, NJ, USA) to the bottom of the PCB. The lens in this setup had 6 mm diameter and 6 mm effective focal length. The beam size of the upwardly directed light was expanded by the lens and delivered as 1 cm diameter circular radiation to the PDMS top surface. An Nd:YAG laser (Minilite I, Amplitude, CA, USA) was used as the pulsed light source, having 532 nm wavelength, 7 ns pulse duration, and 10 Hz pulse repetition frequency. The laser intensity was less than 5 mJ/cm^2^, lower than the safe use limit of 20 mJ/cm^2^ (ANSI Z136.1). Thus, this measurement technique can be applied to the living human body. Note also that a 2D array-based volumetric imaging system has the advantage that no scanning was required.

We conducted an experiment to verify the feasibility of a single-shot PAI method. Ultrasonic signals detected by the CMUT were measured simultaneously on several channels by an oscilloscope (DSOX 2004A, Keysight Technologies, Santa Rosa, CA, USA) with 5000 points, 500 MHz sampling frequency, and 8 averaging acquisition. The data of all elements were automatically obtained by using eight 8-channel multiplexers. The signal data were acquired in a measurement time interval of less than 1 s and processed using a digital band pass filter from 1.5 to 8 MHz, with reference to the detected main frequency of 3.7 MHz. Image reconstruction was performed using the delay and sum method based on the PA effect.

## 3. Results

### 3.1. Photoacoustic Image Results Based on Phantom

**Figure 3:**
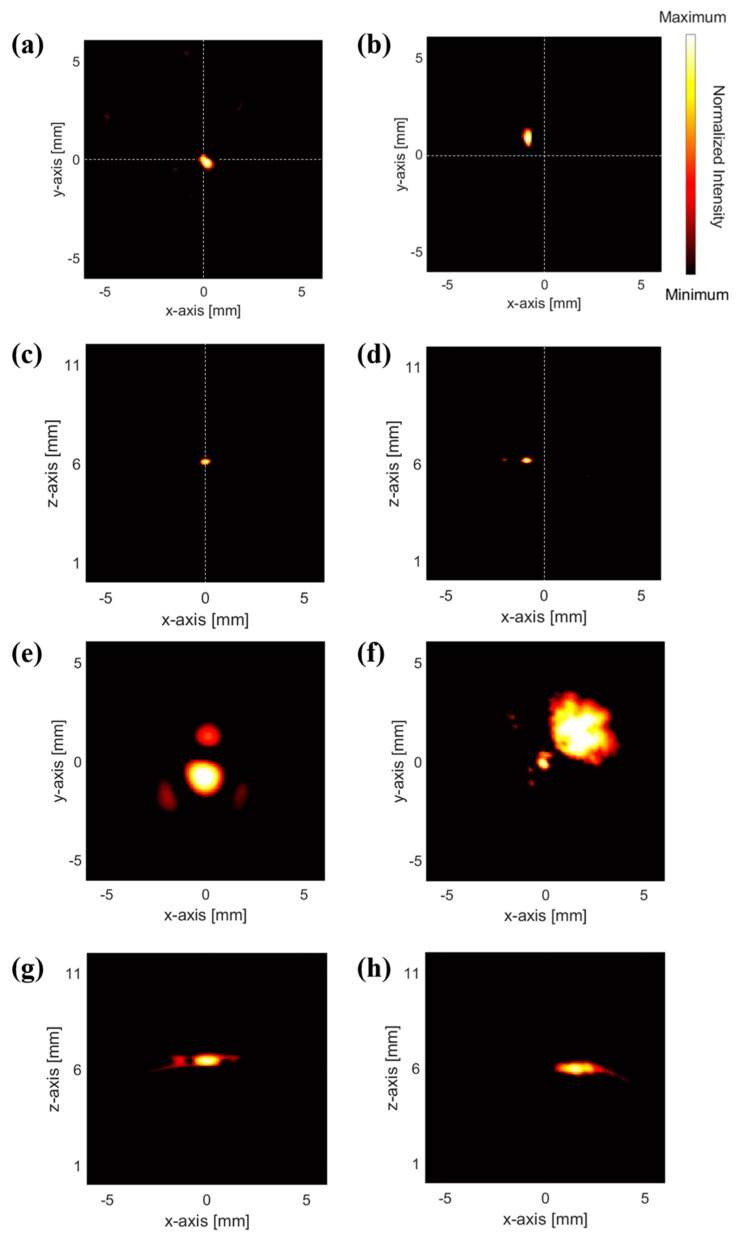
C-mode PA images of 0.3 mm pencil lead, (**a**) on-axis (**b**) off-axis. B-mode PA images of 0.3 mm pencil lead, (**c**) on-axis (**d**) off-axis. C-mode PA images of 2 mm red ink, (**e**) on-axis (**f**) off-axis. B-mode PA images of 2 mm red ink, (**g**) on-axis (**h**) off-axis.

First, we performed PA experiments featuring point targets of pencil lead and red ink with 0.3 and 2 mm diameter, respectively, to estimate spatial resolution and validate the imaging feasibility for both solid and liquid materials. In each case, the target contacted the top of the PDMS. As a result of the geometric nature of the ring array, the target image qualities differed on-axis and off-axis. Thus, the objects were targeted on-axis and off-axis to confirm the reliability of the obtained images (Figure 3). The c-mode and b-mode images in Figure 3 show that this system can perform volumetric imaging. All images consisted of 254 dpi and were obtained in the c-mode plane of the PDMS top surface, which was 6 mm from the CMUT. The results suggest that the ultrasonic waves emitted via the PA effect at the contact area propagated acoustically to the CMUT and could be imaged in the near field. In particular, the image results for the pencil lead phantom show that contact surface imaging is possible in the dry state, as those experiments were conducted with waterless contact. The 2 mm red ink object occupied a larger area, and the on-axis image shape was more accurate than that of the off axis-image. The emitted acoustic signal intensity exhibited differences caused by the light gradient, which was reduced by the axis-effect image contrast and signal-to-background noise ratio. Thus, the on-axis image results featured a more accurate shape because the on-axis signal reduction due to the light gradient was low and all elements had high receiving signal amplitudes. In addition, spatial resolution in the 3 dB bandwidth in this system is estimated to 400 μm by the PSF image.

**Figure 4:**
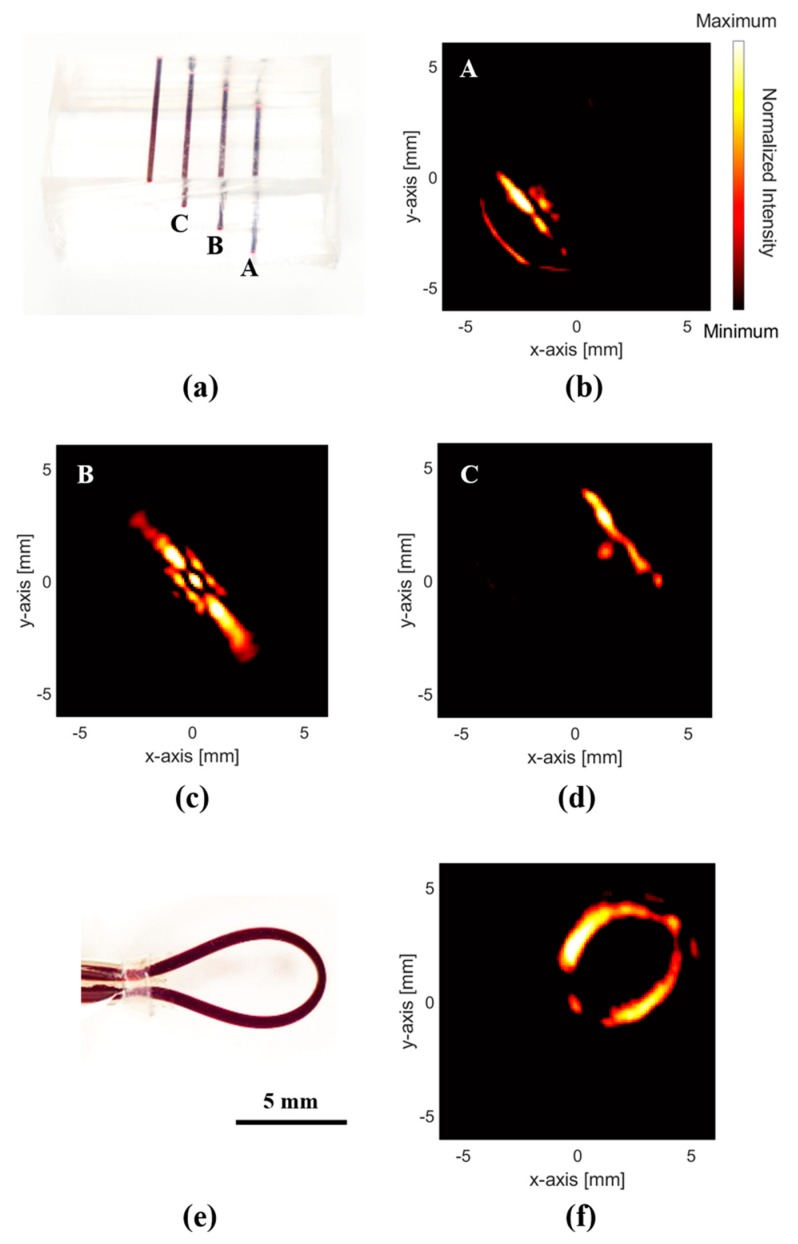
(**a**) Optical images of tube phantoms filled with red ink with different depths and widths. (**b**–**d**) PA images of near, middle, and far depth tubes, respectively. (**e**) Optical image of curved tube phantom filled with red ink. (**f**) PA image of curved tube in (e).

To demonstrate the PA volumetric imaging capability, line-depth and near-field curved imaging experiments were performed. As shown in Figure 4a,e, we used silicone tube phantoms filled with red ink to create line phantoms according to the phantom depth and curvature. The tubes had 1 mm outer diameter and 0.5 mm inner diameter and were molded using PDMS. In the line-depth phantom, each line was formed with 3 mm width and height. Hence, near, middle, and far depth c-mode images were extracted from 7, 10 and 13 mm planes, respectively, from the CMUT, as shown in Figure 4b–d. Each image exhibits artifacts dependent on the depth from the PDMS layer top and location. In particular, Figure 4c clearly shows the effect of the light gradient as well as an artifact occurring at the ring array on-axis. The results of the curved phantom test to obtain near-field images of a more complex object are shown in Figure 4f. The extracted image has the expected tube shape apparent in the optical image (Figure 4e). This result shows that the proposed PA system can detect curved and multi-line objects in a plane located in the near field.

### 3.2. Photoacoustic Image Results on Living Human Body

**Figure 5:**
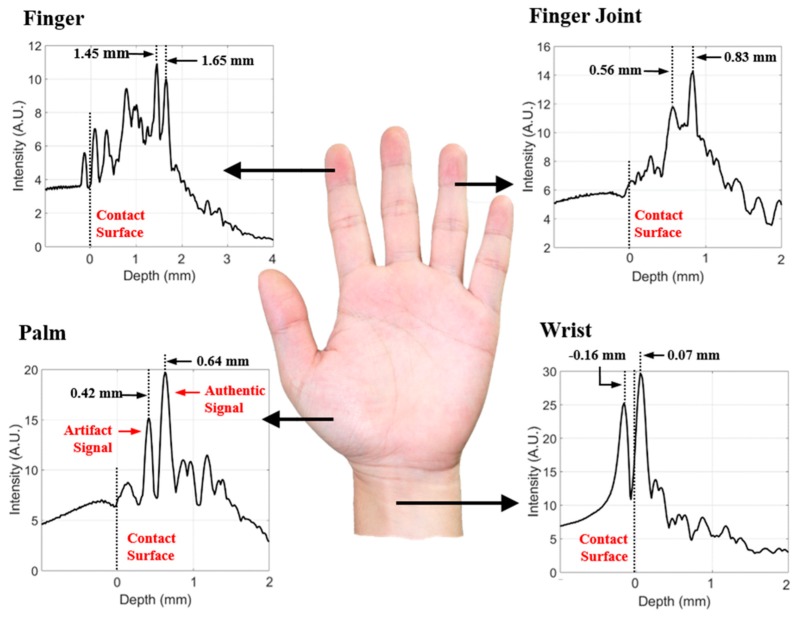
Depth intensity according to body part (finger, finger joint, palm, wrist).

To determine our system’s applicability in biomedicine and biometrics, the living human body was investigated. A 532 nm wavelength with an estimated intensity of less than 5 mJ/cm^2^ was employed, being lower than the safe use limit of 20 mJ/cm^2^. All experiments on a living human body were conducted on the first author (W.Y.C.) with his informed consent. In Figure 5, empirical results obtained from the PAI experiment are displayed according to body part. These graphs implicitly indicate the depth-dependent PA signal intensity. Two peaks indicated by dotted lines appear at certain depths. The first peak is an on-axis artifact generated by the geometric configuration of the ring array, whereas the second peak is the actually detected subject signal. In this experiment, we found that the detected blood vessel depth varies by body part and, also the acoustic signal intensity decreases with increasing depth. Depending on the location of the body part, several PA signals were detected. The main PA signals of the wrist, palm, finger joint, and finger were detected at 0.07, 0.64, 0.83, and 1.65 mm from the skin, respectively. These intensity values show that it is sufficient to measure blood vessels at approximately 1.65 mm depth.

**Figure 6:**
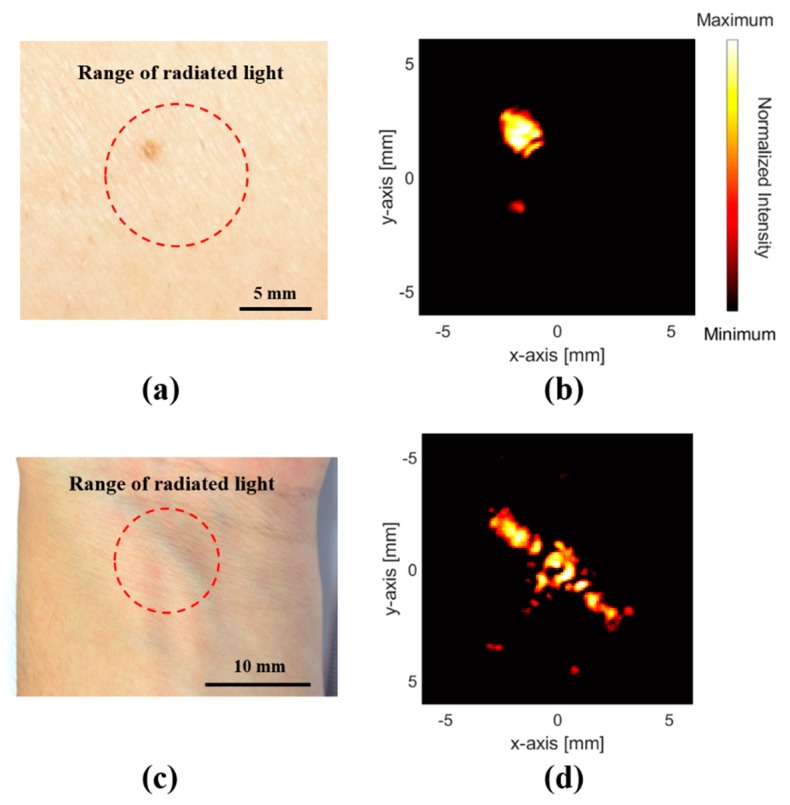
(**a**) Optical and (**b**) PA images of mole in arm. (**c**) Optical and (**d**) PA images of vein in wrist.

Figure 6 presents c-mode images focused on a volunteer’s wrist vein and mole, which can be compared with the accompanying optical images to confirm the living-body image reliability. Thus, the PA mole image of Figure 6b is comparable to the optical image of Figure 6a. The c-mode image was obtained at a 6 mm depth, which was the contact surface position with the PDMS layer top. Hence, the skin and mole were sufficiently distinguishable in this system, as the mole had higher optical absorption and thermal expansion. As regards blood vessel images, the optical and PA images of the wrist vein closest to the skin are shown in Figure 6c,d, respectively. This wrist vein image, as shown in Figure 5, was obtained at a depth of 0.07 mm from the skin.

## 4. Discussion

In the photoacoustic imaging, CMUT is operated in the only reception mode. In receiving mode, CMUT is supplied in very small AC voltage. Since it makes micro current, CMUT has low electrical stress. We measured various phantoms using the system for one month with a 40 V bias voltage and a 10 Hz pulsed laser, and on average, the experiment was conducted for 15 h per week. Any sign of degrading the performance of this system has not been found.

We implemented the single shot photoacoustic imaging system in near field with waterless contact. The PA system is separated into a laser for the transmitting part and an acoustical transducer for the receiving part. This is suitable for near-field imaging because the transmit signal generated by the ultrasonic pulse-echo system does not cause an interference effect on the received transducer. Near field photoacoustic imaging using conventional mechanical scanning has been studied as follows.

- Pulsed photoacoustic signal characterization for near and far-field diffraction effects from 100 kHz to 12.5 MHz. This work presents the measurement result at 8.6 mm distance [22].
- Photoacoustic imaging system applied to various wavelengths in biological tissues from 0.5 to 3 MHz. This work presents the concept of a near-field imaging system, and an imaging result at 17.5 mm depth [23].

Compared to the previous work, our system is competitive with a system that is imaging at 6 mm depth using a receiver with a center frequency of 3.7 MHz.

## 5. Conclusions

This study examined the feasibility of a PA volumetric imaging system for biomedical and biometrics applications. We implemented a single-shot PA system based on a CMUT integrated with electronics, which had wide bandwidth and high receiving sensitivity. The ring array CMUT, which has center frequency of 10.4 MHz in air and 3.7 MHz in PDMS with 6.2 mm array diameter is used. This system is constructed to a 6 mm PDMS matching layer to validate near-field imaging application with a dry contact method. The PA experiment is performed with point targets of solid (pencil lead) and liquid (red ink) phantoms, artificial line-depth vessel phantoms, an artificial curved vessel phantom, and a mole and vein in the living body. We successfully obtained volumetric image results. In addition, a depth-dependent intensity analysis for vessels in the living body was performed.

The proposed system has the following advantages: waterless coupling for dry subject contact, backward-mode laser irradiation for uniform light illumination, a low-profile ultrasonic receiving unit (<1 cm) for the compact device application required for near-field imaging, and short measurement intervals (<1 s) for feasible commercial applications. We expect this technique can be applied to further applications such as vein recognition, under skin diagnosis, and skin disease detection as functional imaging using various wavelengths.
